# Dystrophin Is Required for the Normal Function of the Cardio-Protective K_ATP_ Channel in Cardiomyocytes

**DOI:** 10.1371/journal.pone.0027034

**Published:** 2011-10-31

**Authors:** Laura Graciotti, Jodi Becker, Anna Luisa Granata, Antonio Domenico Procopio, Lino Tessarollo, Gianluca Fulgenzi

**Affiliations:** 1 Department of Clinical and Molecular Sciences, Università Politecnica delle Marche, Ancona, Italy; 2 Neural Development Group, Mouse Cancer Genetics Program, Center for Cancer Research, National Cancer Institute, Frederick, Maryland, United States of America; 3 Center of Clinical Pathology and Innovative Therapy, INRCA, Ancona, Italy; University of Bristol, United Kingdom

## Abstract

Duchenne and Becker muscular dystrophy patients often develop a cardiomyopathy for which the pathogenesis is still unknown. We have employed the murine animal model of Duchenne muscular dystrophy (mdx), which develops a cardiomyopathy that includes some characteristics of the human disease, to study the molecular basis of this pathology. Here we show that the mdx mouse heart has defects consistent with alteration in compounds that regulate energy homeostasis including a marked decrease in creatine-phosphate (PC). In addition, the mdx heart is more susceptible to anoxia than controls. Since the cardio-protective ATP sensitive potassium channel (K_ATP_) complex and PC have been shown to interact we investigated whether deficits in PC levels correlate with other molecular events including K_ATP_ ion channel complex presence, its functionality and interaction with dystrophin. We found that this channel complex is present in the dystrophic cardiac cell membrane but its ability to sense a drop in the intracellular ATP concentration and consequently open is compromised by the absence of dystrophin. We further demonstrate that the creatine kinase muscle isoform (CKm) is displaced from the plasma membrane of the mdx cardiac cells. Considering that CKm is a determinant of K_ATP_ channel complex function we hypothesize that dystrophin acts as a scaffolding protein organizing the K_ATP_ channel complex and the enzymes necessary for its correct functioning. Therefore, the lack of proper functioning of the cardio-protective K_ATP_ system in the mdx cardiomyocytes may be part of the mechanism contributing to development of cardiac disease in dystrophic patients.

## Introduction

Cardiomyopathy is frequently associated with Duchenne and Becker muscular dystrophy. Indeed, with the increased lifespan of the patients bearing such pathologies, cardiac failure is becoming one of the most frequent causes of death [1]. The murine model of Duchenne muscular dystrophy (mdx mutant mouse) bears a point mutation in the gene coding for dystrophin, which causes the premature termination of the polypeptide chain during translation [2], [3]. Consequently, mdx mice lack full-length dystrophin and develop a late onset and progressive cardiomyopathy that has some similarity with that observed in human dystrophic patients [4], [5]. Histological signs of disease in mouse hearts become detectable at 6 months of age with small necrotic and infiltrative foci. Fibrosis develops at later stages, usually by 9 months of age [6]. The biochemical and physiological alterations observed in young and old mdx mice have led to a number of hypotheses to explain the pathogenesis of the cardiomyopathy. These include, altered energetics [7], perturbation in Ca^++^ handling [8], nitric oxide (NO) signaling alterations [9], [10] and increased ROS-mediated damage [11]. Nevertheless, to date there is no evidence of a direct link between dystrophin and any of the aforementioned alterations in the heart.

It should also be noted that physical stress affects the cardiac and skeletal muscles differently in the mdx-related pathogenesis. For example, the heart is a muscle that works continuously, and yet the cardiac disease develops relatively late as compared to the skeletal muscle. Instead, skeletal muscles such as the diaphragm, which are in continuous work, are more affected than other muscles [12]. Nevertheless, even the cardiopathy of the mdx mice, to a certain degree, has been found to worsen with increased workload [13]. These observations suggest that dystrophin may have different roles in skeletal and cardiac muscle. However, it is unclear whether the lack of dystrophin has a primary role in the myopathy by rendering the dystrophic cardiomyocytes more fragile or by affecting the myocyte energetics, which is more relevant when the work-load is increased. We tried to address these questions by studying the energetics of the mdx heart in normal and hypoxic conditions. We found consistent alterations in energy compounds in the mdx heart, including a marked decrease in creatine-phosphate (PC) level under normal oxygenated conditions that become more severe under hypoxic conditions. These data suggest that the mdx heart is more susceptible to an ischemic insult. In searching for clues causing the decrease in PC and increased susceptibility to ischemia we investigated the status of the ATP sensitive potassium channel (K_ATP_) complex since it relies on PC for its correct functioning [14] and it is involved in protection during ischemia [15], [16]. In the heart, there are two main K_ATP_ channel isoforms, Kir6.1 and 6.2. In addition, there are a number of accessory subunits sensitive to sulphonilurea (SUR), the most highly expressed being the SUR2A subunit. It has been shown that the function of the K_ATP_ channel complex, including the Kir6.2 and SUR2A subunit is modulated by intracellular ATP levels. In addition, enzymes such as the creatine kinase muscle isoform (CKm) [14] as well as other glycolytic enzymes [17], [18], [19] are involved in the accurate sensing of intracellular ATP. Specifically, it appears that the close proximity of CKm to the Kir channel complex allows for potent modulation of the channel activity by the CKm substrates PC and creatine, which, respectively causes the channel to close or open.

It has been reported that activation of K_ATP_ is protective against the effect of ischemia [16]. Furthermore, the preconditioning activation of K_ATP_ is responsible for protecting the heart from more severe ischemic insult [15]. Kir6.2 knockout mice bear mild cardiac defects [20] that resemble in part the cardiomyopathy of the mdx mice, being both progressive and late onset; furthermore their heart, like in the mdx mice, is more susceptible to increased workload [21], [22]. These data provided the basis for our investigation into a possible relationship between dystrophin and the Kir6.2 and SUR2A K_ATP_ complex and to studying the K_ATP_ complex activity in the mdx cardiomyocytes. We found a physical interaction of the K_ATP_ channel and CKm with dystrophin, and compromised ATP sensing capabilities by K_ATP_ channels. We hypothesize that the altered function of the K_ATP_ system may be responsible for the enhanced susceptibility of the mdx heart to increased workload and ischemia.

## Results

### Energy compound levels in the mdx heart are severely affected during hypoxia

It has been reported that mdx mutant hearts have metabolic and signaling alterations preceding the development of the cardiomyopathy. These alterations include compromised cardiac contractile function and efficiency, reduced cellular integrity, and exacerbated alterations in mitochondrial citric acid cycle-related parameters and in nutrient signaling pathways related to Akt [7]. Therefore, we decided to further this analysis by testing how mdx hearts respond to stress conditions. To investigate whether energy compound levels in mdx hearts are more affected than in controls during hypoxic conditions we first performed ^31^P NMR in Langendorff-perfused hearts. Both wt and mdx hearts quickly recovered spontaneous beating after the cannulation. In order to obtain homogeneous data, ^31^P NMR spectra recording was initiated 30 minutes after removal of the heart. Data collected at this time point were considered values in normoxic conditions since we used an oxygenated solution for the perfusion. Data collection requires about 10 minutes per sweep and at least 4 complete sweeps were recorded in order to have a sufficient signal to noise ratio. Although this time frame is too long to apply any protocol to induce ischemia or hypoxia that can be reversed without causing any permanent damage, ^31^P NMR spectra-derived quantification of energy compounds is valuable because it provides ex vivo data and it permits a valuable comparison with the data obtained with a different technique. A series of 4 sweeps were averaged to obtain spectra that were then used to quantify the concentration of PC and Pi. ^31^P NMR spectra of wt and mdx mouse hearts show PC/Pi ratio as well as pH values that are significantly altered in mdx mice compared to those of wt (the ratio is 1.11±0.31 in mdx hearts versus 2.04±0.16 p<0.05; in wt; the pH value was 7.03±0.03 versus 7.19±0.03 p<0.02 n = 5 in each experimental group). Representative traces are shown in Fig. S1.

Since ^31^P NMR spectra analysis is not feasible in conjunction with a protocol to induce ischemia we then tested energy compound levels by HPLC on quickly frozen specimens of Langendorff-perfused hearts under normoxic and hypoxic conditions. In normoxic conditions, we confirmed the data obtained by NMR regarding the relative levels of Pi and PC. Specifically, we found a significantly lower PC level in mdx hearts compared to wt (wt  = 36.14±2.13 µmol/g dry weight; mdx  = 26.31±1.48 µmol/g dry weight; p<0.05) whereas the Pi was increased (wt  = 10.66±0.11 µmol/g dry weight; mdx  = 14.83±1.2 µmol/g dry weight; p<0.01; Table 1). Interestingly, under hypoxic conditions, both wt and mdx hearts showed a comparable fall of ATP while ADP, AMP and PC changed differently. Hypoxia caused ADP and AMP concentrations to increase more than two-and four-fold respectively in wt mice. However, in mdx mice, ADP did not increase much while AMP concentration increased seven-fold, which represents a 100% increase when compared to wt (Table 1). Moreover, during hypoxia the PC concentration in wt hearts was reduced to 15% of its value in normoxic condition while in the mdx hearts its concentration was only 9.5% of its normoxic level. These data suggest that the dystrophic hearts are more susceptible to hypoxic insult than wt.

**Table 1:** Data obtained by HPLC analysis of control and mdx mouse hearts.

	Wt		mdx	
	Normoxic	Hypoxic	Normoxic	Hypoxic
PC	36.14±2.13	5.6±0.87	26.31±1.48*	2.45±0.61$
Cr	30.80± 0.84	64.70±6.04	31.00±2.13	56.00±2.08
Pi	10.66±0.11	31.87±3.49	14.83±1.20*	33.10±2.33
ATP	18.25±0.93	8.55±0.32	15.83±0.89	5.85±0.67
ADP	5.80±0.17	12.53±0.58	6.11±0.24	9.10±0.57$
AMP	1.24±0.16	5.28±1.07	1.22±0.16	8.45±0.32$

Normoxic and hypoxic condition are compared. The values represent the compound concentration expressed as µmol/g dry weight ± SE; n = 5 per group.

*t-test p<0.05 significantly different values between mdx and wt in normoxic conditions.

$ p<0.05 by 2 way ANOVA, animal type x condition.

### Loss of function of K_ATP_ in mdx hearts

It has been reported that K_ATP_ functions as an innate mechanism of protection during cardiac adaptation to stress and workload [18]. Therefore we investigated the functional status of the K_ATP_ system in cardiomyocytes isolated from the mdx hearts. We chose to analyze embryonic mdx cardiomyocytes first since we reasoned that adult mdx cardiomyocytes might already be compromised by an advanced dystrophic cardiomyopathy.

Opening of K_ATP_ channels caused by a reduction in the intracellular ATP concentration generates potassium currents that can be studied to directly investigate the functional status of K_ATP_ channels. Therefore, we used three different approaches to reduce the intracellular ATP concentration causing K_ATP_ channel opening.

#### 1) Dialysis of intracellular ATP

**Figure 1:**
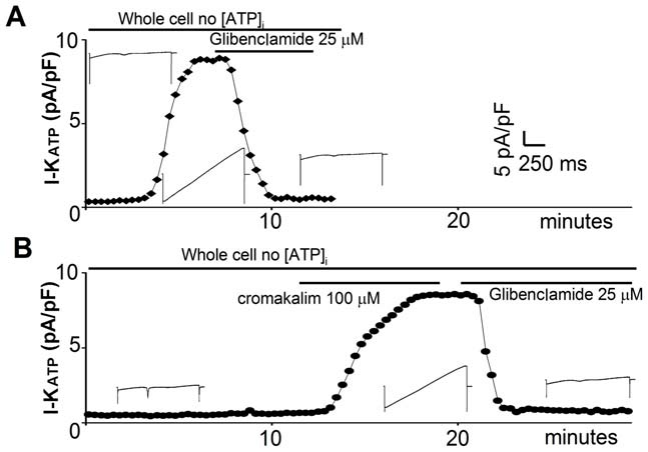
I_ATP_ obtained by dialysis of intracellular ATP is missing in the mdx cardiac cells. Time course of I_ATP_ development in wt (A) and mdx cultured cardiomyocytes (B). In wt cells the dialysis of ATP rapidly induces the I_ATP_ current that is selectively abolished by the application of the antagonist. In the mdx cells no I_ATP_ is induced by the whole-cell recording conditions after 13 minutes. The current develops rapidly after the application of the selective agonist Cromakalim and is rapidly abolished by the selective antagonist Glibenclamide. Insets show the current traces relative to the time point.

We patched the cardiomyocytes to obtain a whole cell configuration using an intracellular solution with no ATP. In wt cardiomyocytes we detected a strong current within 3 to 5 minutes from the membrane break-in (Fig. 1A). This time course is compatible with the dialysis of the physiological intracellular ATP [23], [24]. The current obtained reached its maximum within 10 minutes after the break-in and was completely abolished by 25 µM of the K_ATP_ specific channel blocker Glibenclamide. Furthermore, current leakage at the holding potential of –70 mV inverted its polarity from an inward to an outward current, indicating that the permeability to potassium increased to an extent that the unclamped resting potential of the cell moved closer to the Nernst equilibrium for potassium (i.e. to the potential where potassium is not flowing through the membrane). These data allow the characterization of this current as a K_ATP_ mediated current (I_ATP_). Surprisingly, in mdx cardiomyocytes we were unable to record any I_ATP_ (Fig. 1B).

#### 2) Poisoning of mitochondria

We used the perforated patch clamp method to maintain the physiological intracellular milieu during the recording [25]. Subsequently, we reduced the intracellular ATP level by poisoning the mitochondria with 100 µM dinitrophenol (DNP).

**Figure 2:**
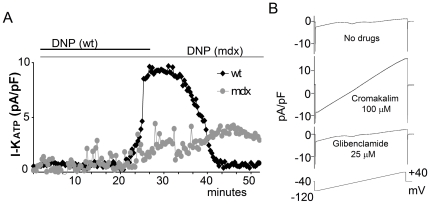
I_ATP_ obtained by poisoning mitochondria is missing in the mdx cardiac cells. A) Time course of I_ATP_ development in wt and mdx cultured cardiomyocytes recorded in perforated patch clamp. In wt cells treatment with DNP poisons the mitochondria, and consequently decreases the [ATP]_i_ leading to the disappearance of the I_ATP_ current by wash out. In the mdx cells no I_ATP_ is induced by DNP treatment after 50 minutes. Longer treatment usually results in membrane instability and cell death. B) In mdx cardiomyocytes in perforated patch clamp the I_ATP_ current develops rapidly after the application of the selective agonist Cromakalim, while the application of the selective antagonist Glibenclamide completely abolishes the current. The lower trace represents the applied voltage protocol.

In wt cardiomyocytes the DNP treatment induces the appearance of a strong potassium current that can be completely abolished by Glibenclamide (25 µM) treatment or washout (Fig. 2A). The DNP-induced current reaches a peak within 5 minutes from its onset. However, DNP treatment of dystrophic cardiomyocytes does not cause any I_ATP_ current (Fig. 2A). Instead, 40–50 minutes after the DNP application the cell membrane becomes very unstable and the seal is lost. Occasionally, some cells are able to withstand the DNP treatment for up to one hour but even these cells do not show any K_ATP_ mediated current, eventually become progressively swollen and form membrane blebs.

#### 3) Treatment with the agonist cromakalim

**Figure 3:**
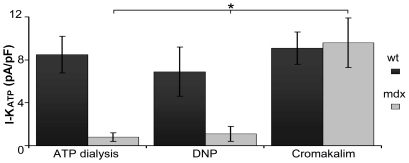
Summary of I_ATP_ density measured at +40 mV. Induction of the I_ATP_ currents by: dialysis of intracellular ATP (n = 8); mitochondria poisoning with DNP (n = 12); or application of the selective agonist Cromakalim (n = 9). Only in the latter case do mdx cardiomyocytes show an I_ATP_ density similar to wt. I_ATP_ in mdx cardiomyocytes is significantly different (* p<0.05) than that induced by ATP dialysis or DNP treatment.

Surprisingly, a strong K_ATP_ mediated current is induced both in the perforated and in the whole cell configuration in mdx cardiomyocytes upon treatment with the potassium channel agonist cromakalim (100 µM) (Fig. 2B, Fig. S2). This current was not different from the one recorded in the wt cells either in its amplitude or in the developing time course. The current obtained was also completely blocked by the antagonist Glibenclamide (25 µM) as in wt cells. The current density in all configurations was quantified at +40 mV and normalized according to cell size (Fig. 3).

Since the agonist cromakalim acts on the K_ATP_ channel auxiliary subunit, these data strongly suggest that the K_ATP_ channels are present and are correctly assembled at the membrane of the mdx cardiomyocyte. However, the cells are unable to sense the changes in intracellular [ATP].

**Figure 4:**
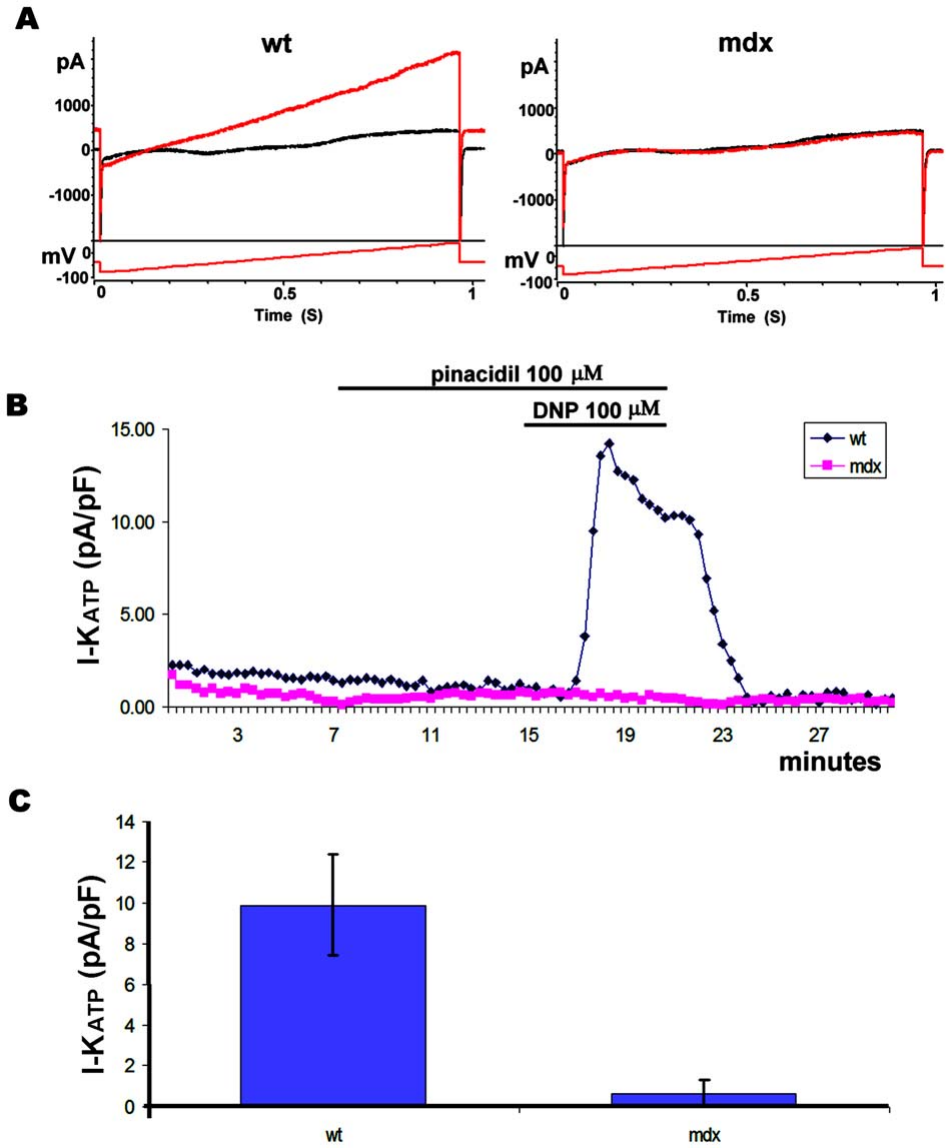
Current recorded from adult cardiomyocytes. A) Wt and mdx traces primed with 100 µM Pinacidil, before and after application of DNP 100 µM in voltage clamp whole cell configuration. The voltage protocol used to elicit the current is also indicated. The red trace shows the current after 5 minutes of DNP application. B) Time course of a recording from wt and mdx cells in relation to the timing of Pinacidil and DNP application. C) K_ATP_ current quantified from the recording of 9 wt (from 3 mice) and 12 mdx (from 4 mice) cells. The current was normalized to the cell size.

To investigate whether the defects observed in embryonic cardiomyocytes were also present in the adult we analyzed the K_ATP_ function in cardiomyocytes freshly isolated from young or adult mice. As shown in Fig. 4 young mdx cardiomyocytes failed to respond to DNP. Cardiomyocytes from old mdx animals also failed to respond to DNP. Total K_ATP_ currents obtained upon perfusion with cromakalim (100 µM) were not significantly different between the old and young cardiomyocytes although we noticed a tendency toward smaller values in the old mdx cardiomyocytes (Table S2). These data suggest that the defect in K_ATP_ function is present at every developmental stage of the mdx cardiomyocytes and is independent of the onset of the histological signs of cardiomyopathy.

### Expression of K_ATP_ isoforms is not altered in mdx cardiomyocytes

To gain insight into the mechanism causing the K_ATP_ channel loss of sensitivity to intracellular ATP level in the mdx cardiomyocytes we investigated the expression level of the different channel isoforms. We performed RT-PCR expression analysis for the principal (Kir6.1, Kir6.2) and the associated subunit (SUR1, SUR2A and SUR2B) isoforms of the K_ATP_ channel in both mdx and wt hearts. We found no changes in the level of expression of any K_ATP_ channel isoform between the wt and dystrophic hearts (Fig. S4). Interestingly, in the ventricular wall of the mouse heart the mRNA for the accessory subunits SUR1, SUR2A and SUR2B are expressed at equal levels while, as previously reported [15], the Kir6.2 subunit is expressed at a higher level than the Kir6.1 subunit (Fig. S4). These data suggest that since there are no imbalances in the level of expression of the different K_ATP_ channel isoforms and subunits, other mechanisms may be causing the K_ATP_ channel loss of sensitivity to intracellular ATP level in the mdx cardiomyocytes.

### Kir6.2 co-immunolocalizes with dystrophin

**Figure 5:**
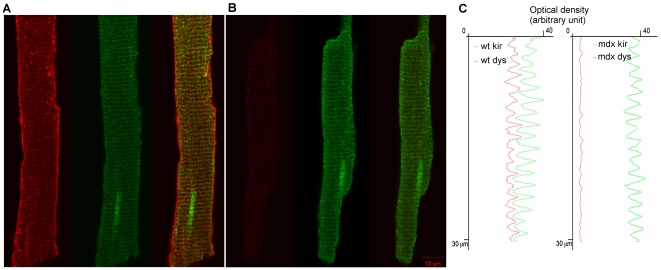
Kir6.2 retains the t-tubule subcellular localization in the absence of dystrophin. Immuno-fluorescence analysis of young adult wt and mdx mutant cardiomyocytes stained for Dys427 and Kir6.2 (A, B). Note dystrophin (red) presence at the plasma membrane and t-tubules of the wt fiber (A, left image) while it is not detectable in the mdx mutant fiber (B, left image). Kir6.2 (green, A and B center images) is associated to the t-tubules in both wt and mdx mutant fibers. A and B, right images, show co-staining of Dys427 and Kir6.2. Longitudinal optical density profiles (C) plotted in red for dystrophin and in green for Kir6.2 show identical spacing in wt cardiomyocytes suggesting co-localization of the two proteins. Note that the profile for Kir6.2 in the mdx mutant fiber is indistinguishable from that of the wt fiber.

Since our electrophysiology data suggest that the K_ATP_ channels are present and functioning in the mdx mutant cardiomyocytes we sought to determine their subcellular localization in isolated control and mutant cardiomyocytes. Immuno-staining of wt cardiomyocytes showed that Kir6.2 localizes in horizontal stripes coinciding with t-tubules, as previously reported [26] (Figure 5). Importantly, Kir6.2 staining of mdx cardiomyocytes revealed a similar striated t-tubule associated pattern. Optical density profiles along the longitudinal axis of the fiber further confirmed the overlapping staining of Kir6.2 with dystrophin in the wt cardiomyocytes. Immunostaining with an anti-dystrophin antibody also showed its presence at the plasma membrane and on the t-tubule as reported by Stevenson et al. and Frank et al. [27], [28]. As expected, dystrophin was not detected in mdx mutant cardiomyocytes. Taken together these data strongly suggest that in wt cardiomyocytes dystrophin and Kir6.2 share the t-tubule localization and in mdx cardiomyocytes Kir6.2 staining is still localized at the t-tubules.

### K_ATP_ and Creatine Kinase co-immuno precipitate with dystrophin

**Figure 6:**
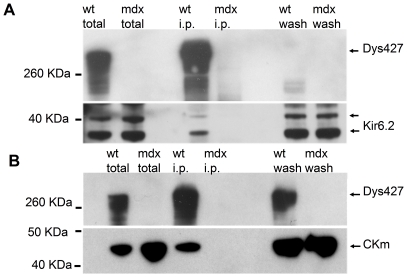
Kir6.2 and CKm co-immunoprecipitate with Dys427. A) Cardiac lysates from wt and mdx animals were immunoprecipitated with an anti Dys427 antibody and probed for the presence of Kir6.2 (lower part). The membrane was then re-probed with the anti Dys427 antibody (upper part). Kir6.2 is abundant in the total lysate of both wt and mdx hearts but co-immunoprecipitates with Dys427 only in the wt sample. B) CKm interacts with dystrophin. Samples as in A were probed with an anti CKm antibody (lower panel) The membrane was subsequently reprobed with an anti Dys427 antibody (upper part). Note that CKm is abundant in the total lysate of both wt and mdx hearts but co-immuno precipitate with Dys427 only in the wt sample.

It has been reported that dystrophin may have scaffolding activity since it mediates the correct membrane localization of several different proteins in specific tissues (reviewed in [29]). Therefore, we investigated whether it may also interact with the K_ATP_ channel complex. We first looked for a physical interaction between dystrophin and Kir6.2, the most highly expressed isoform of the K_ATP_ complex. Cell lysates from mdx and wt cardiomyocytes were immunoprecipitated with an antibody recognizing full-length dystrophin and blotted with an antibody specific for Kir6.2. While the Kir6.2 subunit is present in the cell lysate of both wt and mdx hearts, its presence was detected only in the immunoprecipitation experiment from the wt heart (Fig. 6A, Fig S3).

**Figure 7:**
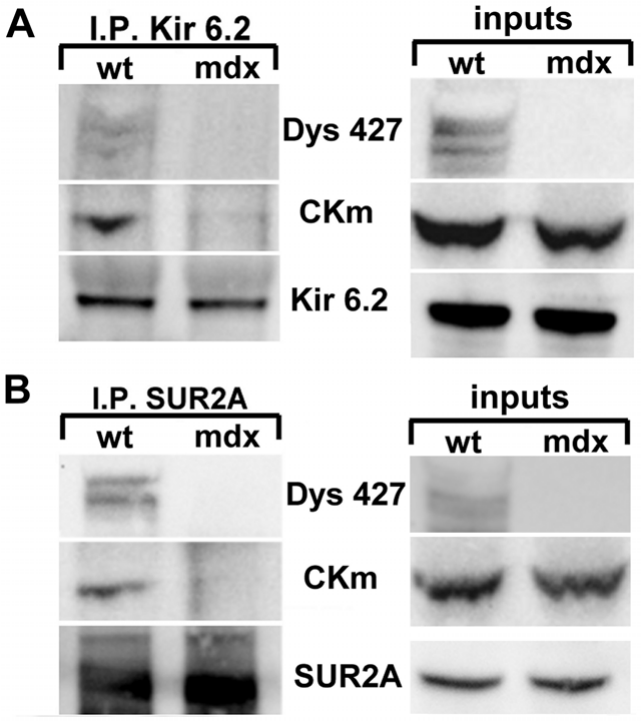
CKm and Dys427 are immunoprecipitated by Kir6.2 and SUR2A. Lysates from wt and mdx mouse hearts were immunoprecipitated with an anti Kir6.2 (A) or SUR2A (B) antibody. Blots were probed with anti CKm, Dys427, Kir6.2 and SUR2A antibodies. Inputs (right panels) show that CKm is present in both wt and mdx lysates but is co-immunoprecipitated by Kir6.2 or SUR2A only in wt lysates where Dys427 is present.

It has been reported that CKm physically associates with K_ATP_ [14] therefore we tested whether CKm could also interact directly or indirectly with dystrophin. Ventricular cell lysates were immuno-precipitated with an anti-dystrophin antibody and the blots were probed with an antibody specific to CKm. Again, as observed for the Kir6.2 subunit CKm was present in the whole cell lysates of both wt and mdx hearts but we detected the presence of CKm only in the IP from the wild type animals (Fig. 6B). The reverse IP was also performed to test whether dystrophin is required for CKm interaction with Kir6.2. Cell lysates from wt and mdx hearts were immunoprecipitated with a specific antibody against Kir6.2 or SUR2A and analyzed for the presence of CKm and Dys427. We found that CKm was immunoprecipitated only in wt lysate (Fig. 7). Importantly, full length dystrophin was also co-IP by Kir6.2 or SUR2A in wt cardiomyocytes. Since the IP was performed in tissues and not in an artificial overexpression system, this result strongly suggests that Kir6.2 and SUR2A do interact directly, or as part of a protein complex with CKm in vivo and that dystrophin is required for these interactions.

### CKm membrane localization is disrupted by lack of dystrophin

**Figure 8:**
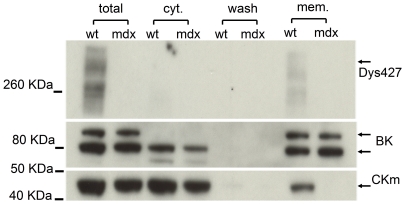
Dystrophin is required for the membrane cellular localization of CKm. mdx and wt whole heart crude lysates were fractionated into the membrane (mem) and cytosolic fraction (cyt) and analyzed by western analysis with antibodies specific for dystrophin (top panel), BK (middle panel) and CKm (bottom panel). Note the presence of CKm in the membrane fraction of only the wt lysate while BK, which was used as a control, is present in both the wt and the mdx samples. The partial degradation for Dys427 is due to the extraction without detergent, required for the membrane separation. Multiple BK bands are due to different glycosylation state of the protein during synthesis.

We have found that CKm is normally present in mdx cardiomyocyte lysates (Fig. 6B) but does not interact directly in vivo with Kir6.2 and SUR2A (Fig. 7). So we investigated whether the loss of dystrophin would affect not only CKm interaction with Kir6.2 and SUR2A but also its subcellular localization. Crude lysates of ventricular wall muscle were separated into the cytosolic and membrane fraction. The fractions were tested by western blot for the presence of CKm, BK, and full length dystrophin (Dys427). The BK potassium channel was used as control for the membrane fraction. As expected, CKm was highly expressed in the cytosolic fraction of ventricular cardiomyocytes from both wt and mdx animals. Surprisingly, while we could detect some CKm in the membrane fraction from wt hearts, no CKm was present in the equivalent fraction from the mdx mice (Fig. 8). These data, together with the co-immunoprecipitation results suggest that dystrophin is essential for the proper localization of CKm at the plasma membrane.

## Discussion

In the present study, we tested the functionality of an enzymatic/ion channel multimeric complex, i.e. the K_ATP_ channel complex in the mdx dystrophic mouse model. The K_ATP_ channels are highly expressed in cardiac tissue, as well as in other metabolically active tissues, where they are believed to be responsible for the fine metabolic modulation of membrane potential-dependent cellular functions [30]. Interestingly, we found that dystrophic mdx hearts have a significant reduction in PC levels in normoxic conditions and are more susceptible to hypoxia than controls. It has been reported that normal K_ATP_ channel function is cardioprotective during an ischemic insult and it relies on the activity of PC and its enzyme CKm. Moreover, a mutation in the gene encoding the SUR2A subunit disrupting the catalytic K_ATP_ channel gating has been linked to dilated cardiomyopathy in human [31]. Thus, we focused on the analysis of the K_ATP_ channel complex function and its regulatory enzymes to gain insight into the pathogenesis of the cardiomyopathy caused by the mdx mutation.

We found that the mdx cardiomyocytes are unable to sense changes in their metabolic state. In addition, we show that specific K_ATP_ channel complex subunits and CKm, an enzyme essential for the ATP sensing capabilities of K_ATP_, co-immunoprecipitate with dystrophin. Importantly, the membrane location of CKm is lost in mdx cardiomyocytes.

We used three different strategies to reduce the intracellular ATP concentration in order to open the K_ATP_ channel. In wild type cardiomyocytes all strategies were successful and produced comparable current values, confirming the presence of functional channels and their ability to sense variations in ATP levels. In the perforated patch clamp configuration there is no dialysis of the intracellular content with the pipette solution and therefore the intracellular enzymatic milieu remains unaltered. With the perforated configuration, DNP-induced chemical ischemia in normal cardiomyocytes caused the insurgence of a potent current that we characterized as K_ATP_-derived current. In fact, this current could be blocked by the specific K_ATP_ channel blocker glibenclamide, and an identical current could be induced by the specific K_ATP_ channel agonist cromakalim. Furthermore, the current was hyperpolarizing and reversed at about −80 mV strongly suggesting that it was driven by potassium ions. Surprisingly, mdx cardiomyocytes recorded in identical conditions did not develop this current upon DNP treatment. These results were further confirmed by recording the mdx cardiomyocytes in whole cell configuration. Strikingly, the K_ATP_ current that quickly developed in normal cells, following a change in intracellular ATP level was never observed in the mdx mutant cells. However, in the mdx cells the current could be recorded in response to the treatment with cromakalim, a specific agonist of the SUR2A component of K_ATP_ [32]. This is important because it provides evidence that the K_ATP_ multi-octameric complex is both expressed and assembled at the membrane in mdx cardiomyocytes. It also suggests that the K_ATP_ channel does not open in response to a decrease in the intracellular concentration of ATP.

The interaction between Kir6.2 and dystrophin is further confirmed by the immunostaining of adult cardiomyocytes for dystrophin and Kir6.2. The normal localization of Kir6.2 in wt cardiomyocytes has been described to be associated with the t-tubule and excluded from the muscle fiber membrane where Kir6.1 channels are present instead (25). We also found Kir6.2 in t-tubules of wt cardiomyocytes. But most importantly, we found that dystrophin and Kir6.2 share the same location on the t-tubule. Moreover, lack of dystrophin does not affect Kir6.2 localization at the t-tubules.

It is not yet clear how the K_ATP_ channels sense changes in the metabolic conditions of the cell. The intracellular ATP/ADP ratio appears to be the most important factor regulating sarcolemmal K_ATP_ channels, whereas ATP and ADP act, respectively as endogenous blockers and openers of the channels (reviewed by [33]). In this regard, it has been suggested that there is a close functional relationship between the adenylate kinase and CKm phospho transfer, defining the directionality of nucleotide exchange within the sarcolemmal K_ATP_-channel vicinity [19]. It has also been shown that CKm is physically associated with the sarcolemmal K_ATP_ channels [14]; genetic disruption of the CKm gene alters the responses of K_ATP_ to metabolic alteration [34] and pharmacological inhibition of CKm reduces the effect of mitochondrial uncoupling on K_ATP_ channel activity [35], thus reinforcing the concept that K_ATP_ requires CKm for correct functioning.

**Figure 9:**
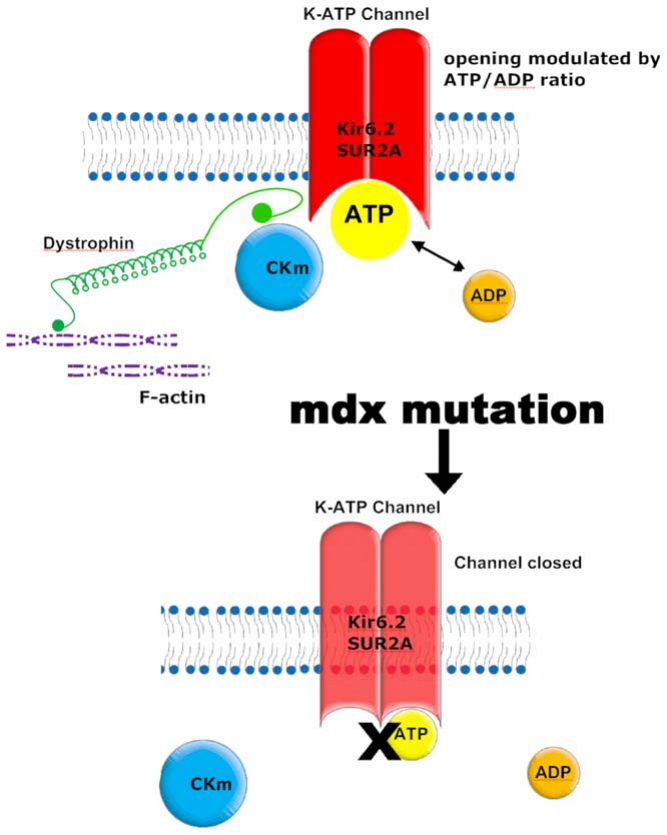
Model of how dystrophin could affect K_ATP_ channel function.

Our co-immunoprecipitation results and the loss of CKm from the mdx membrane fraction suggest that dystrophin may act as a scaffold creating a protein complex where the K_ATP_ and all the accessory proteins may interact and allow a coordinate sensing of the cellular metabolic status (Fig. 9). Moreover, the uncoupling of the K_ATP_ channel from cellular metabolic signals has been shown in another model of cardiac failure. As we found in the mdx heart, the intrinsic biophysical properties of the K_ATP_ channel were unaltered and the functional deficits of the channel were caused from cellular remodeling [36].

The lack of dystrophin has considerably different effects in the cardiac and skeletal muscle. The skeletal muscle is affected soon after birth with continuous cycles of degeneration/regeneration and clear signs of cell damage, while cardiac muscle does not show any cell damage until late adulthood and dilated cardiomyopathy become evident at a very old age [37]. Even then this damage is relatively modest and the main histopathological sign is the gradual accumulation of fibrosis. Despite the lack of obvious histological abnormalities in young mdx mice, alterations in metabolic and electrophysiological properties have been reported [7], [8]. Moreover, the mdx-related cardiomyopathy becomes more pronounced and appears earlier in animals subjected to sustained physical activity [13]. Interestingly, the mdx^5cv^ mutant mouse, despite a much worse skeletal muscle phenotype, shows reduced cardiac abnormalities when compared to the original mdx mutant animal. This is probably a reflection of the reduced cardiac workload associated with the diminished physical activity caused by the more severe skeletal muscle phenotype in the mdx^5cv^ mutant [38]. Further studies have shown that injury to dystrophin-deficient hearts was significantly correlated with the cardiac workload since reduction of the workload after the initial physical stress improved contractility and prevented injury in the mdx hearts in an ex vivo paradigm [39]. Collectively, all these data suggest that the dystrophic hearts are capable of sustaining a moderate workload but they fail when higher performance is required. Now we provide a mechanism to explain how the mdx mutant heart is more susceptible to stress. Our data support the hypothesis that dystrophin functions as a scaffolding protein that correctly assembles the enzymes required by the K_ATP_ channel for sensing the metabolic state of the cell and protecting it from physical stress or ischemia (Fig. 9). This mechanism is further supported by the demonstration that functional K_ATP_ channels are cardioprotective since they are required for adaptation to stress [22]. Any impairment in their physiological function, such as the defects that we have reported in the mdx heart may contribute to the development of cardiomyopathy. This finding is important because it furthers our understanding into the molecular deficits caused by a lack of dystrophin and, if confirmed in human, may provide the basis for the development of new strategies for the pharmacological treatment of DMD cardiomyopathy.

## Materials and Methods

### Animal

Wild type (C57BL/10 ScSn, wt) and genetically dystrophic (C57BL/10 mdx) male mice (Jackson Laboratory) were used for all experiments. Animals were treated in accordance with the guidelines provided by the Animal Care and Use Committee of the National Cancer Institute at Frederick, Maryland and in accordance with guidelines established by the Italian Council for animal care. A total of 8 animals per group (2–3 months old males for the young adult and 12-13 months old for the old adult group) were used for the electrophysiology, 3 animals per group were used for RT-PCR (2–3 month old males; see Methods S1; primer used in table S1) and western blot analysis and 5 animals per group for biochemistry (10–12 months old). Embryos at embryonic day 17.5 (E17.5) were used for deriving embryonic cardiomyocytes.

### Heart isolation and perfusion

Mice were heparinized (500 IU/kg i.p.) 15 minutes prior to the surgical heart explantation and then anesthetized with 50 mg/kg i.p. sodium pentobarbital. The hearts were rapidly excised and perfused using a standard Langendorff method at a constant pressure of 55 mmHg with KHS (118 mM NaCl, 4.7 mM KCl, 2.2 mM CaCl_2_, 1.2 mM MgSO_4_, 0.5 mM EDTA, 25 mM NaHCO_3_, 11 mM glucose, pH 7.3; all reagents are from Sigma). Heart rate and left ventricular pressure were checked using a latex balloon, placed into the left ventricle and connected to a pressure transducer. Only hearts with a heart rate higher than 300 bpm after 30 min of perfusion were used for NMR (see Supporting Information Methods S1) and biochemical analysis (normoxic condition). Hearts were subjected to global hypoxia by switching to a 95% N_2_, 5% CO_2_ saturated perfusion medium for a period of 10 min and used for biochemical analysis.

### Biochemical analysis

High-energy phosphate compounds, creatine and inorganic phosphate (Pi) were estimated by HPLC methods as previously described [40]. Briefly, after perfusion, normoxic and hypoxic hearts were quickly frozen in chilled isopentane and freeze-dried for at least 20 hours. Samples were powdered with a mechanical homogenizer (Mixer Mill, MM200). The dried tissue powder was dissolved in 0.42 M perchloric acid and, after neutralization and precipitation by 1 M KOH, the extract was injected into the HPLC system.

### Cellular protein sub-fraction and Western blotting analysis

Animals were euthanized in a CO_2_ chamber and the heart quickly removed. The ventricular wall was homogenized in ice-cold buffer I (TRIS 10 mM, NaH_2_PO_4_ 20 mM, EDTA 1 mM, protease inhibitor cocktail (Roche) pH 7.8) and centrifuged at 5000 g ×10 min. The supernatant was ultra-centrifuged at 35000 g; the pellet containing the membrane fraction was dissolved in buffer II (HEPES 20 mM, NaCl 150 mM, Triton X100 1%, pH 7.5). Total protein concentration was determined using the Bradford method. The cytosolic and membrane fractions were separated on 4%–12% Tris-Glycine Gels (Invitrogen) and electroblotted onto a polyvinylidene difluoride membrane (Invitrolon). The membrane was divided in three strips by cutting at approximately 110 and 60 KDa. The upper part was used to detect the full length dystrophin (Dys427) with anti-DYS1 antibody (Novocastra Labs), the middle part was used as a control of the cell membrane fraction by hybridizing with an antibody specific to the calcium modulated potassium channel BK (APC-021, Alomone Labs) and the lower part for detection of the CKm (sc-15164, Santa Cruz Biotech). The membrane strips were incubated with the specific primary antibody overnight at 4°C. Appropriate peroxidase conjugated secondary antibody was applied for 1 hour at RT and detected with chemiluminescent reagent (GE HealthCare). The strips were recomposed in the original position and X ray films were exposed and digitized for further data analysis.

### Immunoprecipitation

The ventricular wall was homogenized in ice cold buffer II (GE Healthcare). Pre-clearing was done with goat or rabbit serum (10 µl/ml) and 10 µl of protein A/G PLUS-Agarose (sc-2003) for one hour at 4°C and centrifuged for 10 min at 13500 rpm at 4°C. The supernatant was incubated with an anti-DYS1 (Novocastra Labs; 1/100), an anti Kir6.2 or anti SUR2A (sc-20809, sc-25684 Santa Cruz Biotech 1/200) antibody overnight at 4°C. The resulting immuno complex was precipitated with protein A/G PLUS-Agarose for 3 hours at 4°C. Beads were washed with buffer II (5×500 µl) using micro columns (GE Healthcare), re-suspended in Laemmli sample buffer, boiled for 3 min and the proteins recovered by centrifugation at 13500 rpm ×2 min were analyzed by western blotting as described above with the CKm, Kir6.2 (ab-79582, AbCam 1/1000), SUR2A (sc-32462 Santa Cruz Biotech 1/200) or Dys427 antibodies.

### Electrophysiology

Mouse embryonic cardiomyocytes were cultured from wt and mdx mice and used for electrophysiological measurements after 4 days in vitro. Briefly, embryonic hearts were isolated and cultured by successive digestion with 0.25% trypsin (Gibco) and 0.2% collagenase type II (Sigma). Fibroblast contamination was minimized by pre-plating cells onto tissue culture dishes for 60 minutes. The cardiomyocyte enriched cultures were plated onto laminin-coated (Invitrogen) 1 cm glass coverslips placed on a 6 cm culture dish at a density of 2×10^4^ cells per cm^2^. Growth medium consisted of 25 mM glucose DMEM lacking L-glutamine (Gibco) supplemented with 10% fetal bovine serum (Hyclone) and 1% gentamycin, 1% penicillin/streptomycin, and 1% antimycotic. A single coverslip was transferred into the recording chamber and perfused with Tyrode solution (in mM: NaCl 136.5, KCl 5.4, CaCl_2_ 1.8, MgCl_2_ 0.53, glucose 5.5, HEPES−NaOH 5.5; pH 7.4). Perforated patch clamp was obtained with borosilicate glass pipette filled with (in mM): KCl 140, MgCl_2_ 1, HEPES−KOH 5 (pH 7.3) and Nystatin dissolved in DMSO (0.1 mg/µl) at a final concentation of approximately 400 µg/ml. A ramp from -120 mV to 40 mV in 1 sec was applied every 20 second from a holding potential of −40 mV. I_ATP_ was elicited by different methods: by poisoning of mitochondria with dinitrophenol (DNP; 100 µM), which causes a drop in [ATP]_i_ leading to a potent potassium current [41] within 10 to 20 minutes; with the specific agonist Cromakalim 100 µM, and by dialysis of ATP in whole cell configuration with no ATP in the recording pipette (intracellular as in perforated but without nystatin). Glibenclamide 25 µM (Sigma) and Cromakalim 100 µM (Sigma) were used respectively as antagonist and agonist of I_ATP_. Membrane capacitance, which is directly related to cell size, was calculated for each cell by measuring the area under the transient capacitive currents elicited by 5 mV depolarizing pulses from −40 mV and acquired at a sampling rate of 50 kHz, after subtraction of the steady-state current component. Cell capacitance was used to normalize the current values to the cell size such that the values are expressed as pico Ampere/pico Farad (pA/pF).

### Dissociation and electrophysiological recording of adult cardiomyocytes

Mice were heparinized (500 IU/kg i.p.) 15 minutes prior to the removal of the heart. The hearts were rapidly excised (from mice anesthetized with Fluotane) and perfused using a standard Langendorff method at a constant pressure of 55 mmHg with “Isolation buffer” (130 mM NaCl, 5.4 mM KCl, 0.5 mM MgCl_2_, 25 mM HEPES, 0.33 mM NaH_2_PO_4_, 22 mM D-glucose, 1 mM Lactic acid, 3 mM Pyruvic acid, 5 U insulin, 0.1 mM EGTA pH 7.4; all reagents are from Sigma). After 3–5 min of washing the solution was changed to the “digestion solution” (Isolation solution without EGTA with added 50 mM CaCl_2,_ and Collagenase Type II 300 U/ml Worthington Lakewood NJ). Digestion was performed for 5–8 min and then the heart was minced in Isolation buffer plus 250 mM CaCl_2_, without EGTA. The dissociated cardiomyocytes were centrifuged at 100 g for 5 minutes and resuspended in DMEM at room temperature.

Cardiomyocytes transferred to the recording chamber were perfused with 130 mM NaCl, 5.4 mM KCl, 0.5 mM MgCl_2_, 1 mM CaCl_2_, 5.5 mM HEPES, 5 mM D-glucose. Rod shaped adult cardiomyocytes were recorded in whole cell configuration as described in [5]. The pipette solution was: 55 mM KCl, 85 mM K-Gluconate, 1 mM MgCl_2_, 10 mM HEPES, 100 µM EGTA, pH 7.4 and maintained at a holding potential of −40 mV. DNP and Pinacidil were dissolved in DMSO as stock and dissolved to the final concentration in the perfusion solution just before their use. Ramp voltage protocol (120 mV/s) was applied every 20 s. The K_ATP_ current was evaluated at +40 mV. Glibenclamide (25 µM) completely inhibited the current and was used to pharmacologically characterize the current. Pinacidil (100 µM) was used to prime the K_ATP_ channel such that the subsequent application of DNP 100 µM allowed the channel to open [35].

### Immunostaining of adult cardiomyocytes

Adult cardiomyocytes were dissociated as described above, plated on laminin coated coverslips, fixed with 3.5% paraformaldehyde in PBS for 10 minutes at room temperature and placed in ice cold methanol for 5 minutes on ice. Cardiomyocytes were then rinsed (3×5 min) in PBS and blocked with 5% donkey serum in PBS and 0.1% triton X100 for one hour at RT. Cells were then washed (3×5 min) with wash buffer (PBS with 0.5% donkey serum and 0.01% triton X100). The primary antibody incubation was performed for one hour at RT in wash buffer. Anti Dys-427 (NCL-DYS1, Novocastra 1/1000) and anti Kir6.2 (APC-020, Alomone labs 1/1000) were used to detect the full-length dystrophin and Kir6.2 subunit, respectively. Alexa Fluor 568 donkey anti mouse antibody (1/2500) was used to detect dystrophin; Alexa Fluor 647 donkey anti rabbit was used to detect Kir6.2 (1/2500). The secondary antibody incubation was performed for 30 min at RT in wash buffer. Coverslips were then washed (3×5 min) and mounted for confocal microscopy analysis (Zeiss LSM-510).

Optical density profiles were integrated from a 100 pixel height box in the appropriate axis with image-J and plotted in excel (Microsoft).

### Statistical analysis

Data (mean±SE) were compared by the Student's T test or by the one way ANOVA test where appropriate. P<0.05 was considered statistically significant.

## Supporting Information

Figure S1 **^31^P NMR spectra.** Averages of 4 traces from a control mouse heart (continuous line) and from an mdx mouse heart (dotted line) are shown. Note the increase in the peak area of Pi and the decrease in the peak area of PC in the mdx trace.
(TIF)

Figure S2 **Current recorded from an mdx neonatal cardiomyocyte in voltage clamp whole cell configuration.** The voltage protocol used to elicit the current is also shown. The red trace shows the current after 10 minutes from the break in, and the black trace the current obtained 5 minutes after perfusion with 100 µM of Cromakalim. The red trace shows a fast outward spike of current that arises when the applied ramp reached approximately -50 mV. This current is likely due to the opening of a mixture of calcium and sodium voltage gated channels and is shunted in the black trace by the I_ATP_. The recording conditions are: intracellular solution (in mM): KCl 140, MgCl_2_ 1, HEPES−KOH 5, pH 7.3. Perfusion solution (in mM): NaCl 136.5, KCl 5.4, CaCl_2_ 1.8, MgCl_2_ 0.53, glucose 5.5, HEPES−NaOH 5.5; pH 7.4.
(TIF)

Figure S3 **Kir6.2 and α-Syntrophin but not Rho-GDI co-immunoprecipitate with Dys427.** Heart lysates from wt and mdx animals were immunoprecipitated with an anti Dys427 antibody and analyzed by Western analysis. Membranes were cut horizontally using the molecular markers as reference and probed with an anti- Kir6.2, α -Syntrophin, Rho-GDI and anti Dys427 antibody. Note, that dystrophin is detected as expected in the wt but not in the mdx mutant hearts whereas Kir6.2 and α -Syntrophin (used as positive controls for the IP) are present in the total lysate (inputs) of both wt and mdx heart but co-immuno precipitate with Dys427 only in the wt sample. RhoGDI (used as negative control for the IP) is present in the inputs but does not co-IP with dystrophin.
(TIF)

Figure S4 **The mRNA level for the SUR2A, SUR2B, SUR1, Kir6.1, Kir6.2 and CKm genes is unaltered in the mdx hearts compared to wt controls.** Real time PCR amplification of cDNA obtained from reverse transcription of mRNA extracted from wt and mdx heart. Note the lack of significant differences in expression of any of the tested genes between genotypes.
(TIF)

Table S1 **Primers used for real time PCR.**
(DOC)

Table S2 **I-K_ATP_ induced by the application of agonist (cromakalim 100 µM) and measured at +40 mV.** Values (average and SE are expressed as pA/pF).
(DOC)

Methods S1 **NMR spectroscopy methods.**
(DOC)
