# Supplementary figures and images for: Dystrophin Is Required for the Normal Function of the Cardio-Protective KATP Channel in Cardiomyocytes
Source: PLoS One. 2011 Oct 31;6(10):e27034. doi: 10.1371/journal.pone.0027034 (PMC3205025; doi:10.1371/journal.pone.0027034)

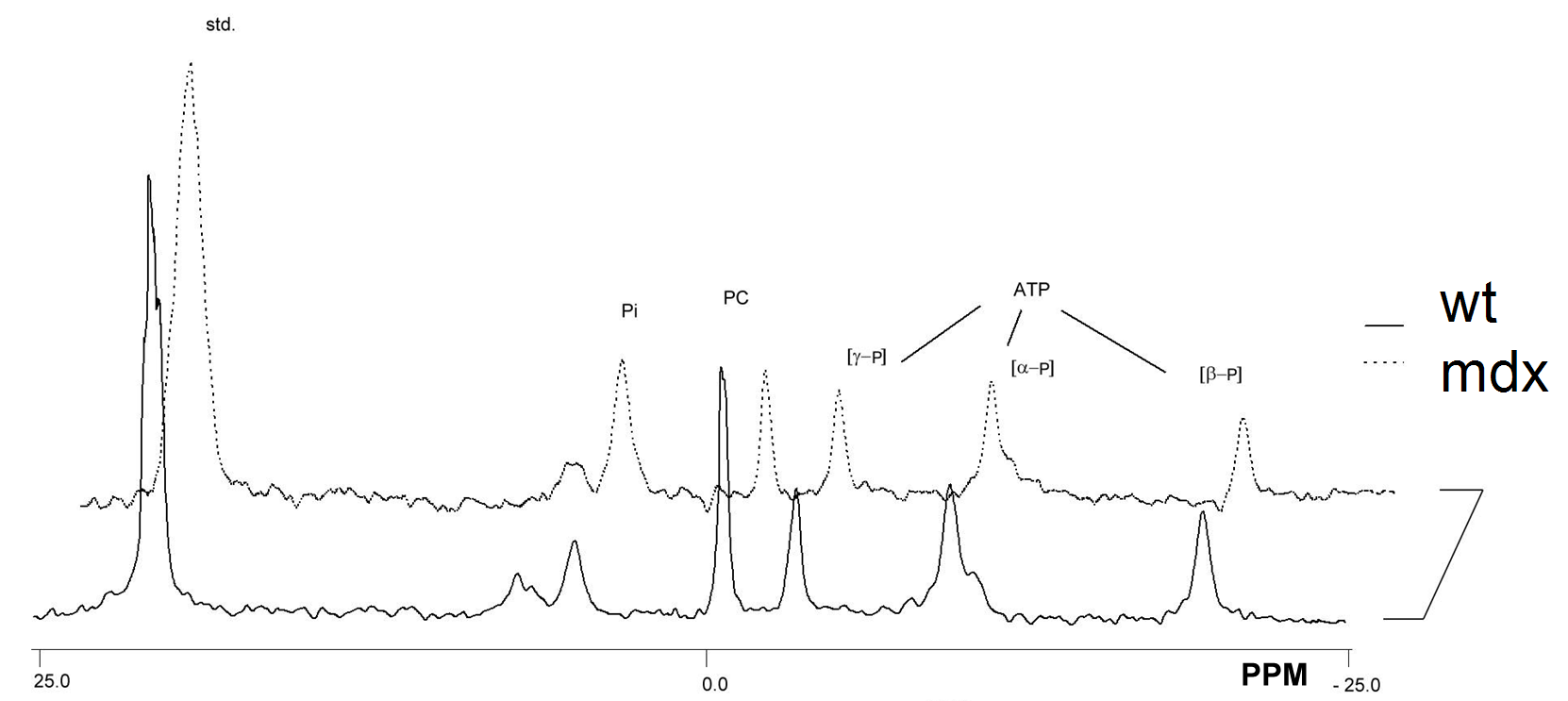

Supplement: Figure S1 — 31P NMR spectra. Averages of 4 traces from a control mouse heart (continuous line) and from an mdx mouse heart (dotted line) are shown. Note the increase in the peak area of Pi and the decrease in the peak area of PC in the mdx trace. (TIF) [file pone.0027034.s001.tif]

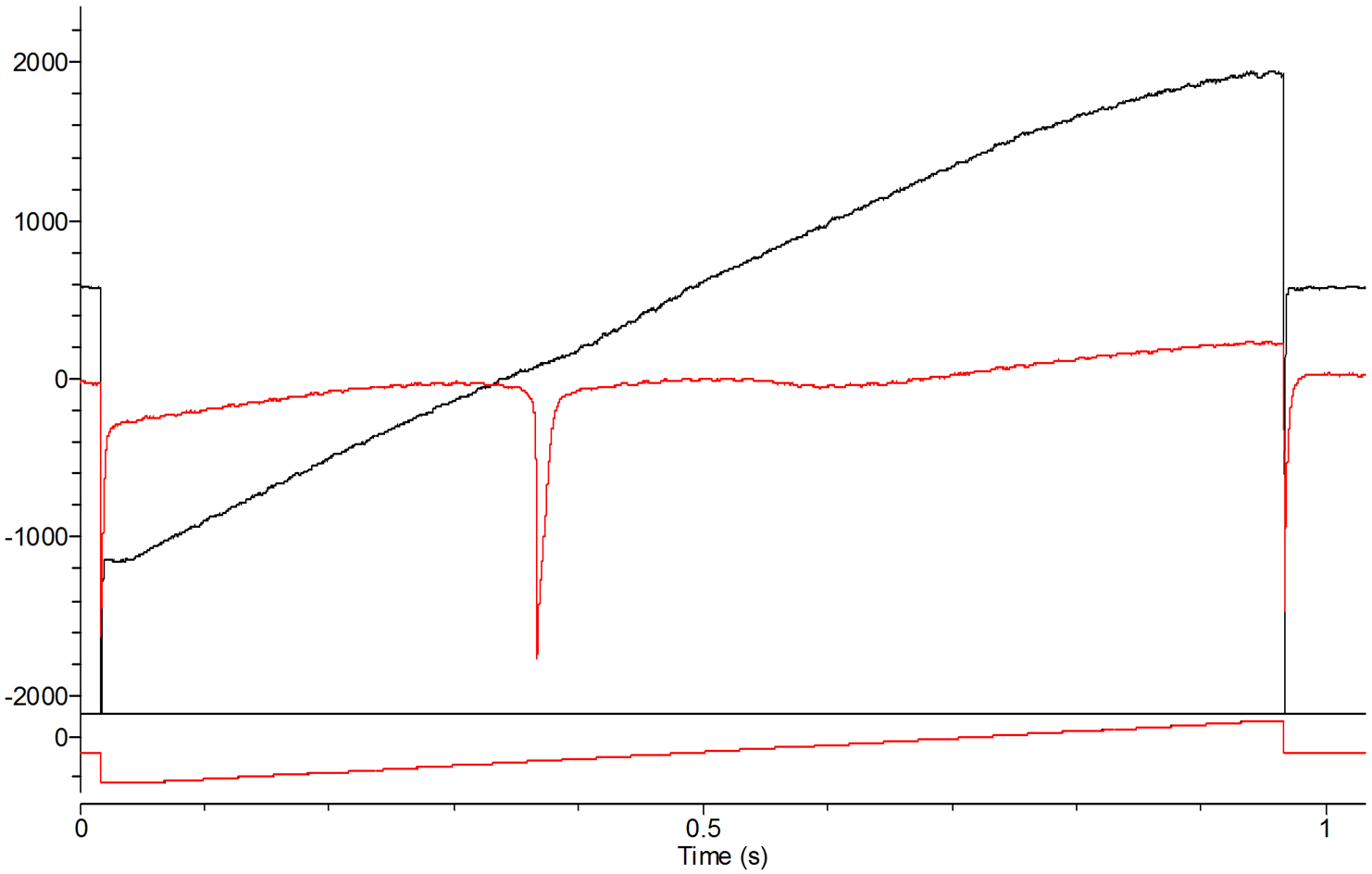

Supplement: Figure S2 — Current recorded from an mdx neonatal cardiomyocyte in voltage clamp whole cell configuration. The voltage protocol used to elicit the current is also shown. The red trace shows the current after 10 minutes from the break in, and the black trace the current obtained 5 minutes after perfusion with 100 µM of Cromakalim. The red trace shows a fast outward spike of current that arises when the applied ramp reached approximately -50 mV. This current is likely due to the opening of a mixture of calcium and sodium voltage gated channels and is shunted in the black trace by the IATP. The recording conditions are: intracellular solution (in mM): KCl 140, MgCl2 1, HEPES−KOH 5, pH 7.3. Perfusion solution (in mM): NaCl 136.5, KCl 5.4, CaCl2 1.8, MgCl2 0.53, glucose 5.5, HEPES−NaOH 5.5; pH 7.4. (TIF) [file pone.0027034.s002.tif]

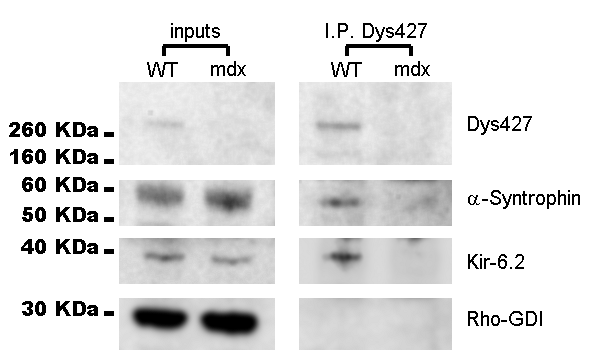

Supplement: Figure S3 — Kir6.2 and α-Syntrophin but not Rho-GDI co-immunoprecipitate with Dys427. Heart lysates from wt and mdx animals were immunoprecipitated with an anti Dys427 antibody and analyzed by Western analysis. Membranes were cut horizontally using the molecular markers as reference and probed with an anti- Kir6.2, α -Syntrophin, Rho-GDI and anti Dys427 antibody. Note, that dystrophin is detected as expected in the wt but not in the mdx mutant hearts whereas Kir6.2 and α -Syntrophin (used as positive controls for the IP) are present in the total lysate (inputs) of both wt and mdx heart but co-immuno precipitate with Dys427 only in the wt sample. RhoGDI (used as negative control for the IP) is present in the inputs but does not co-IP with dystrophin. (TIF) [file pone.0027034.s003.tif]

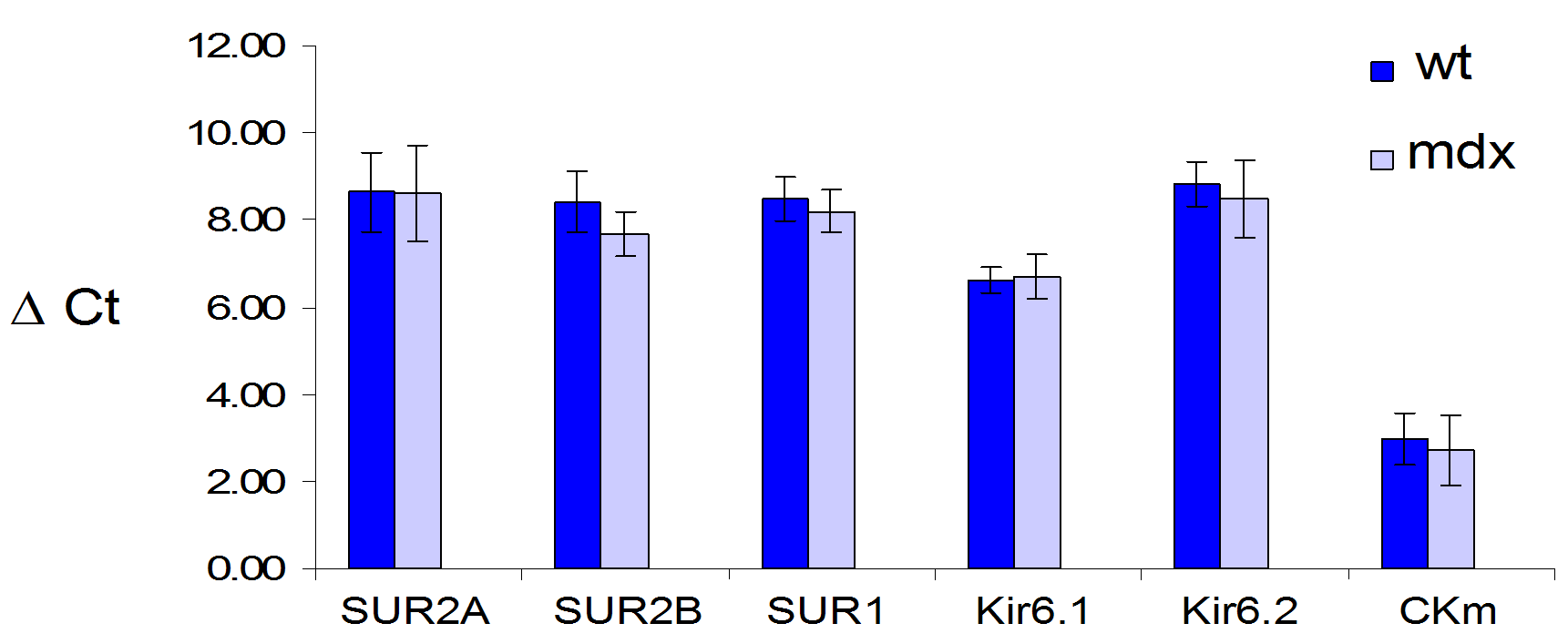

Supplement: Figure S4 — The mRNA level for the SUR2A, SUR2B, SUR1, Kir6.1, Kir6.2 and CKm genes is unaltered in the mdx hearts compared to wt controls. Real time PCR amplification of cDNA obtained from reverse transcription of mRNA extracted from wt and mdx heart. Note the lack of significant differences in expression of any of the tested genes between genotypes. (TIF) [file pone.0027034.s004.tif]
