# Supplementary material for: Dystrophin Is Required for the Normal Function of the Cardio-Protective KATP Channel in Cardiomyocytes
Source: PLoS One. 2011 Oct 31;6(10):e27034. doi: 10.1371/journal.pone.0027034 (PMC3205025; doi:10.1371/journal.pone.0027034)
Supplement: Table S1 — Primers used for real time PCR. (DOC) [file pone.0027034.s005.doc]

|  | **Forward** | **Reverse** |
| --- | --- | --- |
| **SUR 1** | tgaagcagaccaacgagatg | gcaatggggatagctgtgtt |
| **SUR 2A** | atcgcacggtcgtaaccata | ggagcaggtttggaccagta |
| **SUR 2B** | atcgcacggtcgtaaccata | ctttccggggtgtcgtatt |
| **Kir 6.1** | accctccaaaagagcgaact | tggagtcatgaactgcacct |
| **Kir 6.2** | accattaaagtgcccacacc | gatgctaaacttgggcttgg |
| **CK-m** | ggctctgtcctctgggttct | tatttaaggcagggcatgga |
| **B-Actin** | taccacaggcattgtgatgg | tctcagctgtggtggtgaag |

Table S1: Primers used for real time PCR.
