# Supplementary material for: Dystrophin Is Required for the Normal Function of the Cardio-Protective KATP Channel in Cardiomyocytes
Source: PLoS One. 2011 Oct 31;6(10):e27034. doi: 10.1371/journal.pone.0027034 (PMC3205025; doi:10.1371/journal.pone.0027034)
Supplement: Table S2 — I-KATP induced by the application of agonist (cromakalim 100 µM) and measured at +40 mV. Values (average and SE are expressed as pA/pF). (DOC) [file pone.0027034.s006.doc]

|  | WT | mdx |
| --- | --- | --- |
| Young cardiomyocytes  (2-3 months) | 10.3 ± 3.2  (n=4) | 8.7 ± 2.6  (n=4) |
| Old cardiomyocytes  (12-13 months) | 9.2 ± 2.9  (n=3) | 6.2 ± 2.8  (n=3) |

Table S2
