# Supplementary material for: Dystrophin Is Required for the Normal Function of the Cardio-Protective KATP Channel in Cardiomyocytes
Source: PLoS One. 2011 Oct 31;6(10):e27034. doi: 10.1371/journal.pone.0027034 (PMC3205025; doi:10.1371/journal.pone.0027034)
Supplement: Methods S1 — NMR spectroscopy methods. (DOC) [file pone.0027034.s007.doc]

**Supporting Information Methods**

***NMR spectroscopy methods***

^31^P NMR spectra were obtained at 121.49 MHz frequency using a 7 Tesla vertical bore (Advance 300 DMX Bruker, 1 cm probe). Spectra were collected sequentially over 10 min periods and data were averaged from 542 free induction decays (pulse width of 6 µs, a pulse angle of 60^0^ , recycle time of 1 sec, and sweep width of 6068 Hz). Resonance peaks were fitted to a Lorentzian function. Peak areas were calculated using a custom designed computer program. Intracellular pH values were calculated from the chemical shift of the pH-dependent peak of Pi relative to the peak of PC (1).

***Total RNA isolation and cDNA preparation***

Total cellular RNA was isolated from heart tissue using OMNIZOL “Total RNA isolation kit” (Euroclone Ltd, UK). The RNA concentration and purity were determined by Nanodrop ND-1000 spectrophotometer (NanoDrop Technologies Inc, USA). Total RNA (500 ng) was reverse transcribed with Omniscript Reverse Transcriptase (Qiagen Inc.) using random octamers. Resulting cDNA (final volume of 20 μl) was stored at -80°C until the analysis.

***Real-time PCR***

Real-time PCR analyses were performed on a DNA Engine Opticon 4 System (MJ Research; Reno, NV) with the iQ-SYBR(r) Green Supermix (BioRad) premixed PCR reagents. Primers (SI Table 1) were synthesized by Invitrogen Corporation, UK. The PCR conditions were: 95°C for 3 min, then 40 cycles including 10 sec at 95°C (denaturing step), 20 sec. at 60°C (annealing) and 30 sec. at 72°C (synthesis). An automatic melting curve analysis was performed in the Cycler determining the melting points of the products. PCR efficiency was confirmed for all genes to ensure equivalent amplification rates relative to β-actin. The amplification products were run on a 1% agarose gel; water was included as a negative control for each PCR run. The threshold cycles for each amplification product of each sample were normalized against the corresponding β-actin threshold cycles analyzed in the same run and condition (Δ CT). For each group of animals, the mean expression of each gene was compared to each other.

References

1 Taylor DJ et al. (1986) Energetics of human muscle: exercise-induced ATP depletion. *Magn Reson Med* 3:44-54.
